# Hepatic steatosis among people living with HIV in Southern Brazil: prevalence and risk factors

**DOI:** 10.1038/s41598-020-65133-7

**Published:** 2020-05-19

**Authors:** Marina Ferri Pezzini, Hugo Cheinquer, Alexandre de Araujo, Carlos T. Schmidt-Cerski, Eduardo Sprinz, Fernando Herz-Wolff, Julia Poeta

**Affiliations:** 1 Post Graduate Program – Science in Gastroenterology and Hepatology, Hospital de Clínicas de Porto Alegre, Universidade Federal do Rio Grande do Sul, Porto Alegre, RS Brazil; 2 Gastroenterology and Hepatology Division; Hospital de Clínicas de Porto Alegre, Universidade Federal do Rio Grande do Sul, Porto Alegre, RS Brazil; 3 Gastroenterology and Hepatology Division, Hospital de Clínicas de Porto Alegre, Universidade Federal do Rio Grande do Sul, Porto Alegre, RS Brazil; 4 Pathology Division, Hospital de Clínicas de Porto Alegre, Universidade Federal do Rio Grande do Sul, Porto Alegre, RS Brazil; 5 Infectious Disease Service, Hospital de Clínicas de Porto Alegre, Universidade Federal do Rio Grande do Sul, Porto Alegre, RS Brazil; 6 Liver Disease Center, Hospital Moinhos de Vento, Universidade Federal do Rio Grande do Sul, Porto Alegre, RS Brazil; 7 Science Health Institute, Centro Universitário Ritter dos Reis, Universidade Federal do Rio Grande do Sul, Porto Alegre, RS Brazil

**Keywords:** Non-alcoholic fatty liver disease, Non-alcoholic steatohepatitis

## Abstract

Chronic liver disease is an important cause of morbidity and mortality among people living with human immunodeficiency virus (HIV) and is frequently related to non-alcoholic fatty liver disease (NAFLD). The objective is to estimate the prevalence and risk factors of hepatic steatosis among consecutive patients with stable HIV infection on antiretroviral therapy (ART). Also, the use of transient elastography (TE) as a mean to identify a subgroup at risk for non-alcoholic steatohepatitis (NASH) and/or liver fibrosis. HIV infected patients were enrolled between August2016 and February2017. Inclusion criteria: ≥18 years with undetectable HIV viral load. Exclusion criteria: pregnancy; alcohol intake ≥20 g/day and co-infection B or C viruses. Patients underwent ultrasound (US) to diagnose liver steatosis. Significant fibrosis (≥F2) was estimated if at least one of the following were present: APRI > 1.0, FIB4 > 3 and/or liver stiffness ≥7.1kPa. Subjects with TE ≥ 7.1kPa were proposed a liver biopsy and NAFLD Scoring System (NAS) ≥ 3 was considered as diagnosis of NASH. A total of 98 patients were included. Liver steatosis was diagnosed in 31 patients (31.6%) and was independently associated with male gender, BMI, ALT and total bilirubin levels. The prevalence of significant fibrosis assessed by TE, APRI and FIB4 was 26.9%, 6.4% and 3.2%, respectively. Seven patients had a TE result ≥7.1kPa. NASH was found in 5 (83.3%). Among HIV infected patients undergoing ART, almost one third have NAFLD. Neither TE, APRI or FIB4 were able to act as surrogates for significant liver fibrosis. Nevertheless, TE ≥ 7.1kPa was able to accurately select a subgroup of patients at risk for NASH.

## Introduction

Human immunodeficiency virus (HIV) infection is a major public health problem worldwide and is rising in around 50 countries^1^. Latest estimates from 2017 indicate that almost 36.9 million people are infected globally^1^, with around 1.8 million in Latin America^2^ and 860.000 in Brazil^3^. Non-alcoholic fatty liver disease (NAFLD) is currently among one of the most common causes of liver disease, present in around 25% of individuals throughout the world^4^. Interestingly, the prevalence of NAFLD seems to be higher in people living with HIV compared to the general population, probably related to use of antiretroviral therapy (ART) and other prevalent factors such as obesity, dyslipidemia and/or metabolic syndrome^5–11^. NAFLD consists of a broad histopathological spectrum ranging from simple steatosis to nonalcoholic steatohepatitis (NASH), which could lead to cirrhosis and hepatocellular carcinoma^12,13^. Abdominal ultrasound (US) is the most common tool used to identify liver steatosis, however liver biopsy is still required to diagnose NASH^14^. Recently, some authors have advocated the use of serum biomarkers, combined or not with transient elastography (TE), to restrict indication of liver biopsy for patients at higher risk for NASH^11,15–17^. The aim of this study was to evaluate the prevalence and risk factors of hepatic steatosis among stable HIV infected patients undergoing ART. Moreover, use of TE was explored as a mean to identify a subgroup of individuals at risk for NASH and/or liver fibrosis to undergo liver biopsy.

## Materials and methods

### Study population

Stable HIV infected patients undergoing ART were consecutively enrolled between August 2016 and February 2017 at the Human immunodeficiency virus /Acquired Immuno-Deficiency Syndrome (HIV/AIDS) outpatient clinic of Hospital de Clinicas de Porto Alegre (HCPA), a National reference center for HIV management in Southern Brazil. Patients ≥18 years old with undetectable HIV viral load (<50 copies/mL) for at least 12 months were included. Exclusion criteria were the following: pregnancy; alcohol intake above ≥20 g/day and co-infection with hepatitis B or C viruses. All patients provided written informed consent. The Research Ethic Board of HCPA approved the study (study code 16-0312), which was conducted according to the Declaration of Helsinki.

### Data collection

Data was prospectively collected by a single investigator using a standardized questionnaire with demographics, physical activity and alcohol consumption. Most recent laboratory results from the last 24 weeks were obtained from electronic medical records charts. Body mass index (BMI) was calculated as weight (Kg)/height(m)^2^. HIV specific information was also obtained, such as time since diagnosis (years), duration of ART (years), most recent CD4 T-cell count (cells/µL), and current ART. The following data was also collected: impaired fasting glucose (IFG; defined as fasting blood glucose ≥100 and <126 mg/dl, type 2 diabetes mellitus (DM2; defined as fasting blood glucose ≥126 mg/dl and/or treatment with oral antidiabetic drug/insulin)^18^, abnormal blood pressure (defined as blood pressure ≥130/85 mmHg and/or treatment with anti-hypertensive drugs)^19^, lipid profile including total cholesterol (TC), low density lipoprotein (LDL), high density lipoprotein (HDL) and triglycerides (TG); aspartate aminotransferase (AST), alanine aminotransferase (ALT) and total bilirubin (TB). Metabolic syndrome was defined according to National Cholesterol Education Program Adult Treatment Panel III (ATP-III) as the presence of three or more of the following: blood pressure ≥130/85 mmHg; fasting plasma glucose ≥110 mg/dL; waist circumference>102 cm for men or>88 cm for women; HDL cholesterol <40 mg/dL for men or <50 mg/dL for women; and TG ≥ 150 mg/dL^20^.

### Steatosis and fibrosis assessment

All patients underwent abdominal US with an experienced radiologist. Liver steatosis was diagnosed according to previously established criteria^21^. The following non-invasive fibrosis markers were calculated: AST to Platelet Ratio Index (APRI) as [AST (IU/L)/AST upper limit of normal (IU/L)/Platelets (10^9^/L) × 100]^22^ and FIB4 score as [age (years) × AST (IU/L)]/[Platelets (10^9^/L) × ALT (IU/L)]^23^. Cut off values used for significant fibrosis (Metavir ≥ F2) were APRI > 1.0 and FIB-4 > 3.0. Transient elastography was performed by an experienced investigator after at least 4 hours of fasting using FibroScan® (Echosens; Paris, France). The standard M probe was used in all except three patients that required the XL probe due to BMI ≥ 30 Kg/m^2^. A liver stiffness result was considered reliable if the interquartile range (IQR) was <30% and the success rate was ≥70%. Significant fibrosis (Metavir ≥ F2) was estimated using a liver stiffness result cut-off ≥7.1 kPa^7,13^. Steatosis was estimated using a controlled attenuation parameter (CAP) cut-off ≥238 dB/m^7,11^. Subjects with TE ≥ 7.1 kPa were proposed a liver biopsy. Histological evaluation was performed by an expert liver pathologist using the NAFLD Scoring System (NAS), which is a result of the unweighted sum of scores of steatosis (0–3), lobular inflammation (0–3), and hepatocellular ballooning (0–2) ranging between 0–8. A calculated NAS ≥ 3 was considered as diagnosis of NASH^24^.

### Statistical analysis

Statistical tests were performed using The Statistical Package for Social Science version 18.0 (SPSS, Chicago, IL). Continuous variables were expressed as median (IQR), and categorical variables were presented as numbers (percentage). Mann-Whitney’s U test was applied for comparisons of continuous variables between groups. Comparisons between categorical variables were made by the chi-square test or Fisher’s exact test, when appropriate. Poisson regression model was used to identify factors associated with hepatic steatosis and to detect any potential confounders. This model was chosen since it is the most accurate in estimating the prevalence ratio (PR) in cross sectional studies^25^. Only variables with a *p* ≤ 0.10 at univariate analysis were entered in the multivariate model. Associations with a *p* ≤ 0.05 were considered statistically significant.

## Results

A total of 98 HIV infected patients were included. Mean age was 49 ± 11 years and 53 (54.1%) were male. Patients characteristics such as demographics, comorbidities and laboratory results are summarized in Table 1. Fatty liver was detected by ultrasound in 31/98 (31.6%). The most common ART regimen used was Tenofovir (TDF) + Lamivudine (3TC) + Efavirenz (EFV) in 23.4% of the patients, followed by Zidovudine (ZDV) + 3TC + EFV in 20.4%. Triglyceride levels ≥150 mg/dL were detected in 54 (55.1%) patients, total cholesterol levels ≥200 mg/dL in 36 (36.7%), and ALT above the upper limit of normal (ULN) in 23 (23.5%). In univariate analysis, fatty liver was associated with male gender, BMI, IFG/diabetes, metabolic syndrome, triglycerides, LDL, total bilirubin and ALT levels, and non-invasive fibrosis scores (APRI and FIB-4). In multivariate analysis fatty liver was independently associated with male gender, BMI, triglycerides, total bilirubin and ALT levels (Tables 2 and 3).

**Table 1:** Characteristics of the study population (n = 98).

**Characteristics**	**Value**
**Demographics**	
Age (years)	49 ± 11
Gender, male (%)	53 (54.1%)
Ethnicity, white	84 (85.7%)
BMI (Kg/m^2^)	25.45 (23.6–28.2)
Physical activity*	18 (18.4%)
**Comorbidities**	
Hypertension (%)	27 (27.6%)
IFG/Diabetes (%)	35 (35.7%)
Metabolic syndrome (%)	31 (31.6%)
Dyslipidemia	79 (80.6%)
**Laboratory**	
Triglycerides (mg/dl)	156 (118–2–8)
Total cholesterol (mg/dl)	188.6 ± 34
Low density lipoprotein cholesterol (mg/dl)	103.3 ± 30
High density lipoprotein cholesterol (mg/dl)	45.50 (38–55)
Aspartate aminotransferase (U/L)	21 (17–26)
Alanine aminotransferase (U/L)	22 (16–30)
Total bilirubin	0.4 (0.3–1.2)
**HIV**	
Time since HIV (years)	15 (6–20)
Time on ART (years)	11 (5–16)
CD4 count (cells/µL)	657.5 (118–208)

Data were expressed as number (%), mean ± standard deviation (SD) for quantitative variables with normal distribution, or median (IQR) for quantitative variables without normal distribution.

Abbreviations: BMI, body mass index; IGF, impaired fasting glucose; ART, antiretroviral therapy.

*Physical activity was considered above 3 times a week.

**Table 2:** Variables associated with presence/absence of liver steatosis in HIV monoinfected patients.

**Characteristics**	**No steatosis n** = **67**	**Steatosis n** = **31**	**PR (95% Cl)**^**a**^ **Unadjusted**	**P univariate**	**Adjusted PR (95% Cl)**^**a**^	**P multivariate**
**Demographics**						
Age (years)	48 ± 12	51 ± 10	1.02 (0.99–1.04)	0.146		
Gender, male (%)	31 (46.3)	22 (71.0)	2.07 (1.07–4.04)	**0**.**032**	2.99 (1.15–8.45)	**0**.**030**
BMI (Kg/m^2^)*	25.2 (23–27.8)	26.8 (24.9–29.6)	1.08 (1.03–1.13)	**0**.**006**	1.13 (1.02–1.30)	**0**.**032**
Hypertension (%)	19 (28.4)	8 (25.8)	0.91 (0.47–1.79)	0.795		
IFG/Diabetes (%)	19 (28.4)	16 (51.6)	1.92 (1.08–3.40)	**0**.**025**	2.21 (0.79–6.24)	0.130
MS (%)	17 (25.4)	14 (45.2)	1.64 (0.93–2.90)	**0**.**090**	1.20 (0.40–3.50)	0.778
**HIV**						
Time since HIV (years)*	14 (4–2)	16 (9–20)	1.02 (0.98–1.06)	0.310		
Time on ART (years)*	9 (3–17)	13 (7–16)	1.03 (0.99–1.07)	0.128		
CD4 count (cells/µL)*	680 (539–864)	607 (538–828)	1.00 (1.00–1.01)	0.484		

Abbreviations: BMI, body mass index; IFG, impaired fasting glucose; MS, metabolic syndrome; ART, antiretroviral therapy; PR, prevalence ration; CI, confidence interval.

Median (IQR)*.

^a^Prevalence ratio and confidence intervals were estimated using Poisson regression.

**Table 3:** Variables associated with presence/absence of liver steatosis in HIV monoinfected patients.

Characteristics	No steatosis n = 67	Steatosis n = 31	Unadjusted PR (95% Cl)^a^	P univariate	Adjusted PR (95% Cl)^a^	P multivariate
**Labs**						
Triglycerides (mg/dL)*	148 (102–188)	199 (147–243)	1.00 (1.00–1.01)	**<0**.**001**	1.00 (1.00–1.01)	**0**.**012**
Total cholesterol (mg/dL)	189 ± 33	187 ± 38	1.00 (0.90–1.01)	0.709		
LDL cholesterol (mg/dL)	107 ± 31	96 ± 30	1.00 (0.98–1.00)	**0**.**083**		
HDL cholesterol (mg/dL)*	46 (40–60)	42 (35–53)	0.99 (0.95–1.02)	0.352		
AST (U/L)*	21 (17–24)	23 (20–29)	1.01 (0.99–1.04)	0.190		
ALT (U/L)*	22 (15–27)	28 (19–42)	1.02 (1.01–1.03)	**<0**.**001**	1.15 (1.07–1.23)	**0**.**003**
Total bilirubin (mg/dL)*	0.3 (0.3–0.6)	0.7 (0.4–2.1)	1.24 (1.02–1.50)	**0**.**027**	2.02 (1.25–3.45)	**0**.**006**
**Fibrosis markers**						
FIB-4 score*	0.860 (0.630–1.220)	0.910 (0.760–1.460)	1.33 (1.08–1.64)	**0**.**007**		
APRI score*	0.197 (0.154–0.306)	0.256 (0.198–1.385)	3.42 (1.53–7.64)	**0**.**003**		

Median (IQR)*.

^a^Prevalence ratio and confidence intervals were estimated using Poisson regression.

Abbreviations: LDL, low density protein; HDL, high density protein; AST, aspartate aminotransferase; ALT, alanine aminotransferase; FIB-4, fibrosis-4; APRI, AST to platelet ratio index; PR, prevalence ratio; CI, confidence interval.

Among the 31 patients with hepatic steatosis, prevalence of significant fibrosis (Metavir ≥ F2) assessed by serum biomarkers (APRI and FIB-4) was 6.4% and 3.2%, respectively. Transient elastography was performed in 26 of the 31 patients with hepatic steatosis, while five patients refused the procedure. Significant fibrosis (Metavir ≥ F2) assessed by TE was found in 7 (26.9%) patients and was associated with triglyceride levels, FIB-4 score and CAP values (Table 4). Six of these patients agreed to undergo a liver biopsy, which was found compatible with NASH in 5 (83.3%) and with mild liver fibrosis without NASH in one. No biopsied patient had significant fibrosis (Table 5).

**Table 4:** Variables associated with TE ≥ 7.1kPa/TE < 7.1kPa in HIV monoinfected patients.

Characteristics	TE ≥ 7.1 kPa n = 7	TE < 7.1 kPa n = 19	Unadjusted *P* PR (95% Cl)^a^ univariate	
**Demographics**				
Age (years)	50 ± 6	51 ± 12	0.99 (0.94–1.04)	0.694
Sex, male (%)	5 (71.4)	14 (73.7)	0.92 (0.23–3.80)	0.908
BMI (Kg/m^2^)*	27.4 (24.4–32.5)	26.8 (24.7–29.3)	1.07 (0.95–1.19)	0.273
Physical activity^§^ (%)	4 (57.1)	4 (21.1)	3.00 (0.86–10.4)	0.083
Hypertension (%)	2 (28.6)	2 (10.5)	2.20 (0.63–7.65)	0.215
IFG/Diabetes (%)	4 (57.1)	9 (47.4)	1.33 (0.37–4.82)	0.661
Metabolic syndrome (%)	5 (71.4)	5 (26.3)	4.00 (0.95–16.8)	0.059
**HIV**				
Time since HIV (years)*	20 (9–22)	16 (6–21)	1.05 (0.95–1.17)	0.353
Time on ART (years)*	14 (7–22)	14 (6–16)	1.05 (0.95–1.15)	0.367
CD4 count (cells/µL)*	698 (541–828)	607 (450–879)	1.00 (1.00–1.00)	0.499
**Labs**				
Triglycerides (mg/dL)*	240 (219–438)	175 (125–228)	1.00 (1.00–1.10)	**0**.**037**
Total cholesterol (mg/dL)	190 ± 36	185 ± 36	1.00 (1.00–1.02)	0.773
LDL cholesterol (mg/dL)	88 ± 27	100 ± 26	0.99 (0.96–1.01)	0.328
HDL cholesterol (mg/dL)*	36 (31–53)	45 (37–51)	0.97 (1.00–1.05)	0.481
AST (U/L)*	26 (20–35)	24 (21–29)	1.02 (0.95–1.10)	0.566
ALT (U/L)*	44 (28–73)	27 (20–40)	1.01 (0.99–1.04)	0.302
Total bilirubin (mg/dL)*	0.7 (0.4–2.2)	0.7 (0.3–2.5)	0.98 (0.56–1.60)	0.938
**Noninvasive markers**				
APRI score*	0.255 (0.149–0.447)	0.274 (0.201–0.401)	1.45 (0.15–14.34)	0.752
FIB4 score*	0.98 (0.66–2.64)	0.90 (0.76–1.0)	1.46 (1.15–1.86)	**0**.**002**
**Transient elastography**				
CAP*	360 (320–364)	272 (246–294)	1.02 (1.00–1.04)	**0**.**049**
CAP ≥ 238 (dB/m) (%)	4 (57.1)	16 (84.2)	0.60 (1.00–3.72)	0.583

Abbreviations: TE, transient elastography; BMI, body mass index; IFG, impaired fasting glucose; ART, antiretroviral therapy; LDL, low density lipoprotein; HDL, high density lipoprotein; AST, aspartate aminotransferase; ALT, alanine aminotransferase; FIB-4, fibrosis-4; APRI, AST to platelet ratio index; CAP, controlled attenuation parameter; PR, prevalence ration; CI, confidence interval.

Median (IQR)*.

^§^Physical activity was considered above 3 times a week.

^a^Prevalence ratio and confidence intervals were estimated using Poisson regression.

**Table 5:** Clinical characteristics of HIV patients with liver steatosis and TE ≥ 7.1 kPa underwent liver biopsy.

Characteristics	Case 1	Case 2	Case 3	Case 4	Case 5	Case 6
**Demographics**						
Age/gender	43/male	44/male	54/male	58/male	44/male	57/female
**Non invasive markers**						
APRI score	0.326	1.013	0.447	0.255	0.243	0.15
FIB4 score	0.66	2.64	1.46	0.85	0.63	0.98
**TE parameters**						
Liver stiffness (kPa)	8.6	9.9	7.6	12	7.9	9.1
CAP	364	320	360	-	231	375
**Biopsy**						
Fragment size (mm)	1.6 ×0.1 ×0.1	1.5 ×0.1 ×0.1	1.7 ×0.1 ×0.1	1.6 ×0.1 ×0.1	1.3 ×0.1 ×0.1	1.6 ×0.1 ×0.1
Histologic diagnosis	NASH Fibrosis F0	Without NASH Fibrosis F1	NASH Fibrosis F0	NASH Fibrosis F0	NASH Fibrosis F1	NASH Fibrosis F1

Abbreviations: FIB-4, fibrosis-4; APRI, AST to platelet ratio index; CAP, controlled attenuation parameter; NASH, nonalcoholic steatohepatitis.

## Discussion

The present study investigated the prevalence and associated risk factors for liver steatosis among stable HIV infected outpatients in a tertiary care center in Southern Brazil. Furthermore, we explored the role of TE to identify a subgroup of patients at risk for NASH and/or liver fibrosis among this population. Using abdominal US, hepatic steatosis was detected in almost a third of our sample. This finding is similar to the prevalence reported in a recent systematic review and meta-analysis conducted among HIV monoinfected patients from several countries worldwide, which found NAFLD using mainly US imaging in 35.3% (95% CI 28.8–42.5)^26^. This prevalence seems to be at least 10% higher than what has been usually reported in the general population. Indeed, a recent meta-analytic assessment of the global NAFLD epidemiology among a large group of unselected individuals detected liver steatosis in only 25.2% (95% CI 22.1–28.6)^4^. Nevertheless, one could assume that the true prevalence of NAFLD is probably higher, since abdominal US is known to detect liver steatosis only when more than 20–30% of hepatocytes are affected^23^. In our study, hepatic steatosis was independently associated with male gender and BMI, as well as TG, ALT and TB levels. Similar results were reported by several other authors^7,8,11,13,22^. Noteworthy, our study did not show an association between NAFLD and HIV parameters such as duration of infection, duration of ART and CD4 cell count. Same results were reported in other studies^8,11^.

Among our group of 26 patients with steatosis that underwent TE, 7 (26.9%) had liver stiffness above 7.1kPa. This result is comparable with that found by other authors that used TE cut-offs between 7.0 and 7.4 kPa as a surrogate for significant fibrosis among HIV infected patients, and found 15% to 27.3% individuals within that range^5,7,23^. Remarkably, none of our six patients with liver stiffness above 7.1kPa that underwent liver biopsy had significant fibrosis. This finding suggests that TE may overestimate fibrosis staging in HIV patients with hepatic steatosis detected by US. On the other hand, all except one of our biopsied patients had NASH, indicating that TE was capable of identifying a subgroup at risk for this condition among HIV patients with liver steatosis. Future studies could explore different cut-off values for TE in this population in order to improve its usefulness as a tool to detect not only NASH, but also significant fibrosis.

Interestingly, there was discordance in our sample between TE and APRI and FIB-4. Indeed, the majority of the patients with TE ≥ 7.1kPa had normal FIB-4 and APRI values. This observation was also reported by other authors and highlights the difficulty of assessing correctly the liver fibrosis staging in this population without the use of an invasive method such as a liver biopsy^5,22^.

Our study has some limitations: it is a cross sectional study, so it was not possible to define the consecutive steps leading to the development of both steatosis and fibrosis in HIV infected patients; only a few of the NAFLD patients agreed to undergo liver biopsy, so we cannot rule out the occurrence of NASH and/or significant fibrosis among the remaining patients that refused the procedure; NAFLD fibrosis score (NFS) was not calculated due to the fact that some variables used for its calculation were not routinely collected for most of the patients that comprised this cohort. Finally, the small sample size among subjects submitted to TE prevented us to further explore multivariate analysis in this subgroup of patients.

In conclusion, it is important to realize that TE using a cut-off of 7.1 kPa was able to accurately detect patients with high risk for NASH among HIV monoinfected individuals with NAFLD. The significance of this finding should be validated in larger cohorts, preferably with long term follow-up. If the ability of TE to correctly identify NASH in this population is confirmed, invasive procedures such as liver biopsy could be reserved for identification of significant fibrosis, since TE was not accurate enough to identify this condition. Further studies should explore different TE cut-offs and/or combinations of non-invasive methods to better select patients at risk for significant fibrosis, because this individuals are the ones at higher risk for progressive liver disease and are the best candidates for more aggressive medical management to avoid deleterious outcomes.
